# The Transcriptome of Rhabdomyosarcoma Cells Infected with Cytolytic and Non-Cytolytic Variants of Coxsackievirus B2 *Ohio-1*

**DOI:** 10.1371/journal.pone.0164548

**Published:** 2016-10-19

**Authors:** Anna Sävneby, Johannes Luthman, Fabian Nordenskjöld, Björn Andersson, A. Michael Lindberg

**Affiliations:** 1 Department of Chemistry and Biomedical Sciences, Linnaeus University, Kalmar, Sweden; 2 Department of Cell and Molecular Biology, Karolinska Institutet, Stockholm, Sweden; The Scripps Research Institute, UNITED STATES

## Abstract

The transcriptomes of cells infected with lytic and non-lytic variants of coxsackievirus B2 *Ohio-1* (CVB2O) were analyzed using next generation sequencing. This approach was selected with the purpose of elucidating the effects of lytic and non-lytic viruses on host cell transcription. Total RNA was extracted from infected cells and sequenced. The resulting reads were subsequently mapped against the human and CVB2O genomes. The amount of intracellular RNA was measured, indicating lower proportions of human RNA in the cells infected with the lytic virus compared to the non-lytic virus after 48 hours. This may be explained by reduced activity of the cellular transcription/translation machinery in lytic enteroviral replication due to activities of the enteroviral proteases 2A and/or 3C. Furthermore, differential expression in the cells infected with the two virus variants was identified and a number of transcripts were singled out as possible answers to the question of how the viruses interact with the host cells, resulting in lytic or non-lytic infections.

## Introduction

Coxsackievirus B (CVB) belongs to the *Enterovirus B* species of the *Picornaviridae* family and has been associated with a wide range of diseases from mild respiratory infections to more severe infections of the central nervous system, heart and pancreas [1]. An enterovirus is a small non-enveloped virus of approximately 30 nm diameter with an icosahedral protein capsid shell. Four structural proteins, VP1-4, form the capsid, where parts of VP1-3 are exposed on the surface while VP4 is on the inside of the capsid. The genome is a single stranded RNA molecule of roughly 7500 nucleotides, with a single open reading frame [2].

Picornaviruses are generally easy to propagate in cell culture and commonly cause cell lysis. However, this is not always the case and persistent, non-cytolytic infections, have been observed both *in vivo* and *in vitro* for a number of picornaviruses, e.g. poliovirus, Theiler´s murine encephalomyelitis virus, foot-and-mouth disease virus, CVB3, CVB4 and CVB5 [3–10].

Regardless of persistent or lytic infection, the picornavirus replication cycle is dependent on an intricate interplay between the viral proteins and genome as well as the proteins of the host cell. Cellular proteins are involved in all stages of the replication cycle and the interactions of the viral and cellular proteins are of great importance [2]. Reports show that the interaction between the virus and host cell apoptotic mechanisms may be a determinant of whether the outcome of an infection is cytolytic or not [11–13]. Several picornaviral proteins, for example VP1, VP2 and VP3 are known to induce apoptotic responses [14–17].

Like other RNA viruses, picornaviruses have high mutation rates and in addition to this they frequently recombine. These factors, along with rapid replication cycles and large volumes of viral progeny, lead to a very diverse population. This heterogeneous population is often referred to as quasispecies and makes the virus easily adaptable [18–20].

The prototype strain CVB2 *Ohio-1* (CVB2O) replicates non-cytolytically in human rhabdomyosarcoma (RD) cells. Previously, CVB2O was adapted to cytolytic replication in RD cells [21], and it was observed that the entry process was facilitated by a hitherto unknown receptor. It was also shown that the change to a cytolytic infection was due to a single mutation in the VP1 protein, a glutamine (Q) to a lysine (K), and that the cytolytic virus triggered apoptotic responses. Furthermore, it was shown that the two virus variants, despite the difference in replication strategy, had very similar growth kinetics, and the infections produced approximately the same amount of infectious units [17].

We have here used differential RNA expression to identify potential host factors that may be involved in the switch to a cytolytic infection and investigated whether there is a difference in the amount of viral RNA present in cells infected with the non-cytolytic (CVB2Owt) and cytolytic (vVP1Q164K) variants of the same virus. The results are important as they provide multiple candidates for further functional studies.

## Materials and Methods

### Infection

RD cells were maintained in Dulbecco’s modified Eagle’s Medium (Sigma-Aldrich) supplemented with 10% newborn calf serum, 2 mM L-glutamine, 100 U/ml penicillin and 0.1 mg/ml streptomycin at 37°C, 5% CO_2_.

RD (ATCC CCL-136) cells were infected in triplicate in four different conditions, resulting in a total of 12 samples. The conditions were: infection with the variant vVP1Q164K and incubation for either 24 hours or 48 hours; infection with CVB2Owt and incubation for 48 hours; and mock-infection (treated identically but without the addition of viruses) and incubation for 48 hours. As in the previous study [17], infections were carried out with multiplicity of infection (MOI) 1, calculated based on tissue culture infectivity dose 50% (TCID_50_) performed on green monkey kidney (GMK) cells. The cells were infected through incubation with the virus, diluted in a serum-free version of the cell culture medium described above, at room temperature for one hour. After the incubation, the supernatants were removed, fresh medium added and the cells were incubated at 37°C, 5% CO_2_ until the selected time points.

### RNA Extraction and Sequencing

At selected time points the RNA was extracted from the cells using RNeasy Mini Kit (Qiagen) according to the manufacturer’s specifications. Briefly, the cells were washed with PBS and detached from the culturing flask by trypsinization. The trypsin was neutralized with serum and the cells spun down at 300 x g for 5 min, after which the supernatant was removed. The cells were lysed with the RLT buffer and homogenized through the use of QIAshredder spin column (Qiagen). 70% ethanol was added and the sample was loaded on the RNeasy spin column and successively washed with RW1 and RPE buffer. The RNA was eluted with 50 μl nuclease-free water.

A cDNA library was prepared from the RNA samples. Sequences were clustered using Illumina cBot and sequenced on HiSeq2500 (HiSeq Control Software 2.0.12.0/RTA 1.17.21.3) with a 2x101 setup in HighOutput mode. Bcl to Fastq conversion was performed using bcl2Fastq v1.8.3 from the CASAVA software suite. The quality scale is Sanger / phred33 / Illumina 1.8+. The transcriptome data reported in this publication have been deposited in NCBI’s Gene Expression Omnibus [22] and are accessible through GEO series accession number GSE69205.

The infections were repeated once more. At the decided time points the supernatant was removed and a corresponding volume of fresh medium was added. The infected cells were then freeze-thawed to release the viral particles and RNA inside, after which the viral RNA was extracted using QIAamp Viral RNA Mini Kit (Qiagen). The extracted RNA was reverse transcribed using random hexamers and the TaqMan reverse transcriptase kit (Applied Biosystem) according to the manufacturer’s protocol. Real-time PCR was performed using an ABI Prism 7500 SDS machine (Applied Biosystems) and the reverse-transcribed cDNA was quantified using SYBR Green master mix (Applied Biosystem) with CVB2 sequence specific primers (EnteroFw, 5GCCCCTGAATGCGGCTAAT3 and EnteroRev, 5ATTGTCACCATAAGCAGCCA3 [23]) according to the manufacturer’s instructions.

### Data analysis

The fragments from 12 different samples were sequenced on the Illumina HiSeq2500. Sequence reads were trimmed using Nesoni clip [24], with adaptors as well as beginnings and ends of quality lower than Q10 (1 error per 10 bases) being removed.

Subsequently, TopHat [25] and the Cufflinks [26] set of tools were used for sequence analysis. With TopHat, the trimmed reads were aligned separately to the human genome, release GRCh37 [27], and the virus genome of CVB2Owt (GenBank accession number AF081485.1). Using Cufflinks, reads were assembled into transcripts whose expression was quantified; with Cuffcompare and Cuffdiff, component tools of Cufflinks, expression levels were compared across samples and conditions using a cutoff of alpha = 0.01.

## Results

### Infection

RNA was extracted from the infected cells at 24 and 48 hours post infection for vVP1Q164K and at 48 hours post infection for CVB2Owt and mock infected cells. At 48 hours there were some signs of cell lysis for cultures infected with vVP1Q164K, but not for the other cultures (data not shown).

### Next generation sequencing reads

On average, 17.31 million reads were detected in each sample, and the total number of fragments was 207.7 million. The fragments were sequenced as paired-ends with, on average, 101 bp read length. The mean quality score was 34.75 with 88.46% of bases Q30 (1 error per 1000 bases).

### Comparisons of intracellular RNA

**Fig 1:**
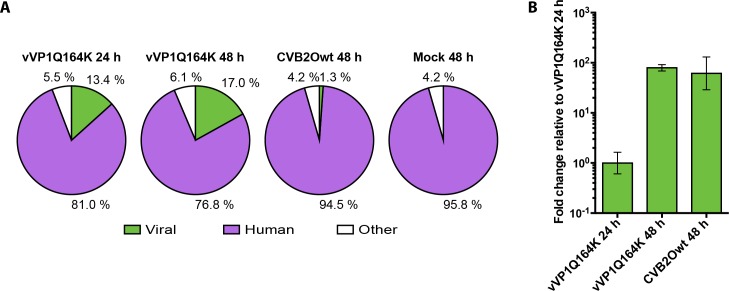
Relative amounts of intracellular RNA. (A) All sequences were mapped against the CVB2O and human genome to obtain the proportions of viral to human RNA in each sample. Sequences that did not match either genome are denoted *other*. (B) The intracellular amounts of viral RNA have been normalized against the amount in the first sample of the triplicate of the cells infected with vVP1Q164K and incubated for 24 hours. Error bars indicate 95% confidence interval.

All sequences obtained were mapped against the human and CVB2O genomes. For every sample the relative amount mapped to each genome was calculated (Fig 1A). In all four conditions 4.2–6.1% were not mapped to either of the genomes. In the cells infected with vVP1Q164K and incubated for 24 hours, 13.4% of the sequences were viral, while 81.0% were human. After 48 hours of incubation the portion of viral sequences had increased to 17.0%. In contrast, out of the sequences obtained from the CVB2Owt-infected cells at 48 hours, only 1.3% were viral, while 94.5% were of human origin. No viral sequences were found in the mock-infected cells.

The amount of intracellular viral RNA, measured with real-time PCR, in the cells infected with vVP1Q164K, increases from 24 to 48 hours (Fig 1B). When instead comparing amounts of viral RNA in the cells infected with the two virus variants at 48 hours, there is no significant difference.

### Differentially expressed transcripts

**Fig 2:**
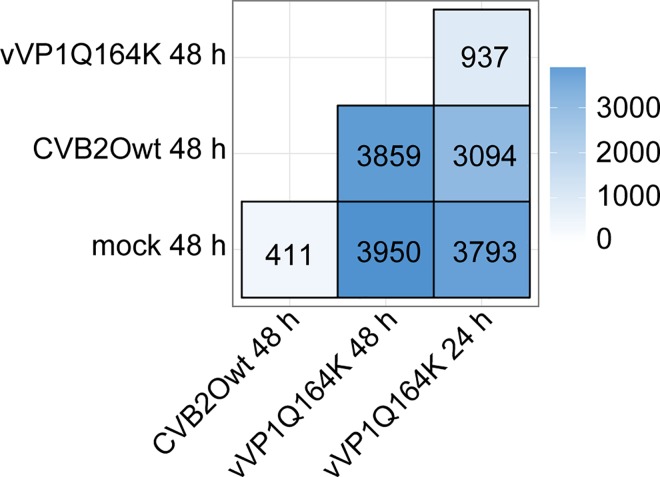
Differentially expressed transcripts at alpha 0.01. The number of transcripts that are differentially expressed compared to the other conditions.

The mapping of the sequences to the human genome showed a total of 34764 transcribed sequences in the cells. Some of these (9014) were not expressed under all of the compared conditions (mock-infected, or infected with CVB2Owt or vVP1Q164K). Out of the 34764 transcribed sequences, 6029 were found to be differentially expressed, as compared to mock cells or other infected cells. In total there were 16044 instances of differential expression (**Fig 2**).

Only 411 transcripts were differentially expressed in CVB2Owt compared to mock. This is sharply contrasted with comparisons of vVP1Q164K (48 hours post infection) and mock, where almost ten times as many (3950) transcripts were differentially expressed. The comparison of vVP1Q164K and CVB2Owt give a similar amount (3859) of differentially expressed transcript as the comparison of vVP1Q164K and mock. The vVP1Q164K sample taken after 24 hours of incubation had fewer differences than the one taken at 48 hours, as compared to the other conditions. When comparing vVP1Q164K infections at the two time points, 937 transcripts were found to be expressed at significantly different levels.

### Differential expression for each sample

**Fig 3:**
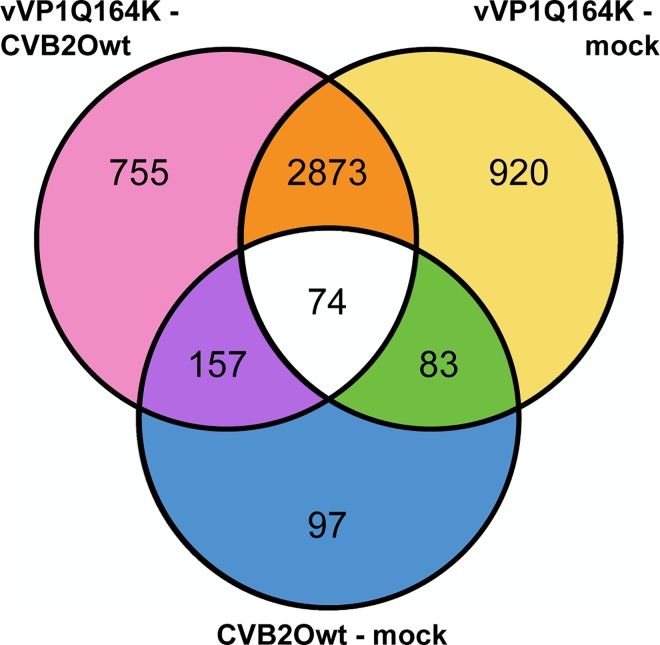
Comparison of differentially expressed transcribed sequences. Comparison of the differences in expression between vVP1Q164K, CVB2Owt and mock, at 48 hours post infection. Difference in expression that (only) occurs in: (pink) vVP1Q164K compared to CVB2Owt; (orange) vVP1Q164K compared to both CVB2Owt and mock; (yellow) vVP1Q164K compared to mock; (purple) CVB2Owt compared to both vVP1Q164K and mock; (white) between all conditions; (green) mock compared to both vVP1Q164K and CVB2Owt; (blue) CVB2Owt compared to mock.

By making comparisons of the differential expressions found in **Fig 1,** for the samples collected at 48 hours post infection, the transcribed sequences were further sorted into the comparisons in which they can be found (**Fig 3**). Of the transcripts found to be differentially expressed, 755 were unique in the comparison between vVP1Q164K and CVB2Owt, that is, for those sequences the virus-containing conditions were significantly different compared to each other, but not to mock. For the comparisons of vVP1Q164K and mock, and CVB2Owt and mock, the corresponding numbers were 920 and 97, respectively. Other changes in expression were unique to one of the conditions, for example, 2873 transcripts were either up or down regulated in the vVP1Q164K sample compared to both CVB2Owt and mock. For CVB2Owt that number was 157, and 83 transcripts were significantly different compared to both the virus-infected conditions. For 74 transcripts the expression differed significantly between all three conditions.

### The most differentially expressed transcribed sequences

**Fig 4:**
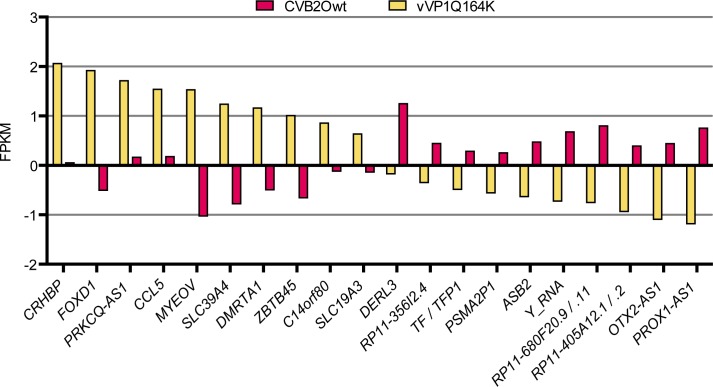
The 20 transcripts most differentially expressed between vVP1Q164K (48 h) and CVB2Owt. The conditions are normalized to mock-cell expression levels.

For further investigation into what facilitates the difference in persistent and lytic virus infections, the 20 most differentially expressed transcripts under the vVP1Q164K (48 h) and CVB2Owt conditions were analyzed. The FPKM (fragments per kilobase of exon per million fragments mapped) value for the conditions for each of these transcripts were normalized against the mock value and plotted in a graph (**Fig 4**). According to the Ensembl database [28], most of these transcripts are protein-coding, the others are pseudogenes or non-coding RNA-genes.

**Fig 5:**
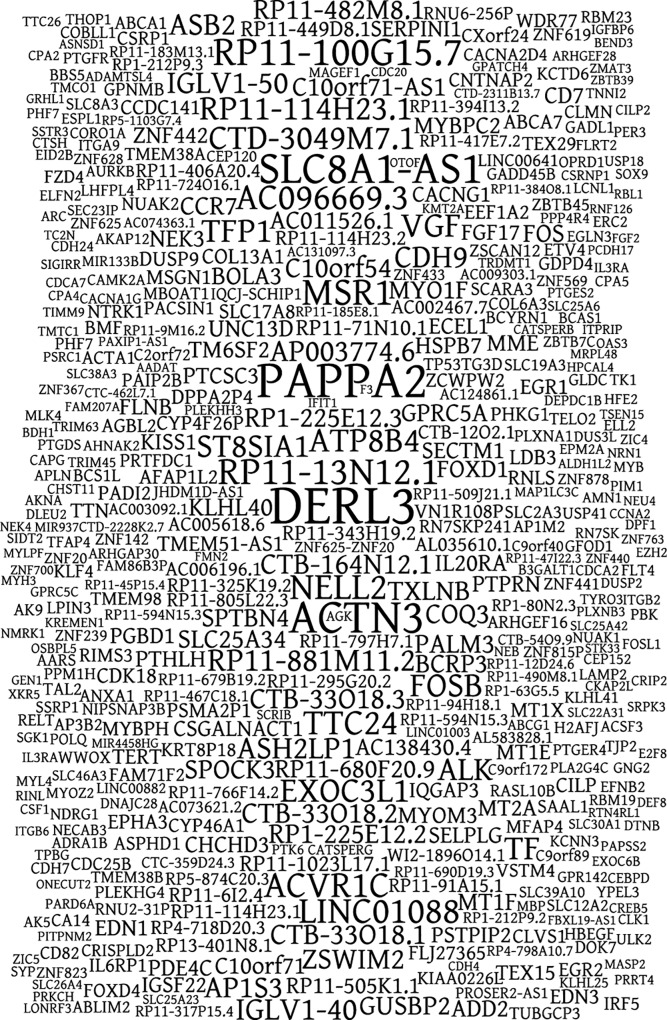
Gene cloud displaying the 500 most significantly differentially expressed transcripts. Font size is proportional to the highest change in expression and inversely proportional to the likelihood of the result.

The 500 most significantly different and with the highest expression levels, out of the 6029 differentially expressed transcripts, were arranged in a gene cloud (**Fig 5**) to emphasize transcripts of interest. The three most differentially expressed transcripts are *DERL3*, *PAPPA2*, and *ACTN3*, coding for proteins derlin-3, pappalysin-2, and the F-actin cross-linking protein, respectively.

## Discussion

The data presented in this study (Fig 1A), show a marked difference in the proportions of viral RNA to human RNA, when comparing the cells infected with the two virus variants that result in persistence versus cytolysis in RD cells. At 48 hours after infection only 1.3% of all RNA sequences in the CVB2Owt-infected cells were of viral origin while at the same time-point 17.0% of the RNA sequences in the cells infected with vVP1Q164K were of viral origin. When measuring only the viral RNA in the cells no difference can be detected between the two virus variants at 48 hours post infection (Fig 1B). These results indicate that there is a difference in the amount of human RNA in the two types of infections. A lower amount of cellular RNA in the RD cells infected with vVP1Q164K might be explained by a general mechanism employed by the viral proteins 2A and 3C to reduce or inhibit cellular transcription and translation as previously has been described for a number of lytic enteroviruses [29]. When the genome of a picornavirus is translated the host cell ribosomes bind to structures in the 5’ untranslated region to initiate translation. The viral genome lacks a cap sequence and the attachment is cap-independent. However, most cellular mRNAs are translated in a cap-dependent manner and some viruses can block or inhibit this translation through cleaving of the eukaryotic initiation factor (eIF) 4G or the poly(A)-binding protein (PABP). The cleavage is most often performed by the viral proteins 2A or 3C [29].

To the best of our knowledge, this is the first time a study has been performed comparing the transcriptome of cells infected with a lytic and a non-lytic variant of the same virus. As expected, the two conditions of vVP1Q164K (24 and 48 hours) show a low number of transcribed sequences with significant difference in expression. The differences that exist between these two conditions could be due to the increased stress in the cells incubated for 48 hours, approaching apoptosis. The comparison with the least number of altered transcript levels was the CVB2Owt-infected cells compared to mock-infected cells. This may be a consequence of a lower degree of stress since no apoptosis is initiated in these conditions and the general structure of RD cells infected with the persistence causing CVB2Owt seems to be unchanged compared to uninfected cells [17].

Little is known about many of the transcripts that have been singled out as the most differentially expressed. The transcripts found in **Fig 4** comprised 12 protein-coding genes, 3 pseudogenes, and 8 non-coding RNA-genes, and the three most differentially expressed transcripts over all (**Fig 5**) were protein-coding. Many, if not most, of these transcripts probably have nothing to do with causing the change in from persistent to lytic infection; however, this is a good starting point when investigating the difference in host cell interaction of persistent and cytolytic virus variants.

*DERL3* was the transcript that stood out as one of the most differentially expressed, both in the comparison of expression in vVP1Q164K and CVB2Owt (**Fig 4**) and in the 500 most differentially expressed transcripts (**Fig 5**). It was up regulated in the CVB2Owt-infection, with the levels of expression for vVP1Q164K being very similar to those of mock. *DERL3* codes for derlin-3, which is a membrane protein that is involved in the Endoplasmic-reticulum-associated protein degradation (ERAD) pathway, where it transports misfolded proteins out of the endoplasmic reticulum [30]. Further studies are needed to ascertain whether this protein transporter has the ability to transfer virions across membranes. Furthermore, it must be determined if derlin-3 is localized to other membranes than those of the endoplasmic reticulum.

It is possible that the cause of the change in persistent to lytic infectionis not induced by the most extreme changes in expression levels. It is also possible that the amino acid change (giving an extra positive charge) in the VP1 protein prevents binding that facilitates the release that occurs with CVB2Owt. Further studies will be required to elucidate the difference in persistent and lytic infections; although it is possible that the transcripts of significance do not display large differences in expression, the natural starting point is to study the transcripts exhibiting the largest differences in expression.

In conclusion, this is the first time whole-genome difference in expression has been studied in cells infected with a lytic and a non-lytic variant of a picornavirus. Comparisons of RNA amounts indicate that the lytic infection cause reduction in the transcription/translation activities of cellular genes as has been observed previously during cytolytic replication of several enteroviruses. Furthermore, there is a marked difference in the levels of transcribed sequences of the infected cells, and we have identified transcripts that might be part of the explanation. However, further work is required to identify the specific mechanism that enables CVB2Owt to cause persistent infections, while vVP1Q164K cause lytic infections.

## Abbreviations

CVB: coxsackievirus B
CVB2O: coxsackievirus B2 *Ohio-1*
ERAD: Endoplasmic-reticulum-associated protein degradation
FPKM: fragments per kilobase of exon per million fragments mapped
GMK: green monkey kidney
MOI: multiplicity of infection
RD: rhabdomyosarcoma
TCID50: tissue culture infectivity dose 50%
